# Effect of Cutting Fluid on Machined Surface Integrity and Corrosion Property of Nickel Based Superalloy

**DOI:** 10.3390/ma16020843

**Published:** 2023-01-15

**Authors:** Shiqi Chen, Pei Yan, Junyi Zhu, Yubin Wang, Wenxiang Zhao, Li Jiao, Xibin Wang

**Affiliations:** Key Laboratory of Fundamental Science for Advanced Machining, Beijing Institute of Technology, Beijing 100081, China

**Keywords:** cutting fluid, Nickel based superalloy, surface integrity, precision machining, corrosion property, electrochemical characteristic

## Abstract

Superalloy parts place high demands on machined surface integrity and serviceability. In the machining process of superalloys, the cutting fluid is usually used to improve the machining performance. Cutting fluids with cooling and lubrication functions have a relatively large effect on the surface microstructure and residual stress as well. The corrosion damage caused by cutting fluid to the machined surface, during machining and residual, are also worth considering. In this paper, the machining performance of typical binary Ni Cr solid solution, age-hardened, nickel-based superalloy NiCr20TiAl T6, under two commonly used cutting fluids, Blasocut and E709, was analyzed, including cutting performance, surface quality, machining surface corrosion characteristics, and so on. The results showed that the surface residual stress could be improved by adding both cutting fluids compared with the deionized water. Blasocut had better lubrication properties, which could reduce friction and heat production. Pitting holes were found on the polished surface after 45 days with E709 cutting fluid, which was more corrosive than Blasocut. According to this research, a reasonable cutting fluid can be selected to reduce the surface corrosion and improve the service life and performance of parts.

## 1. Introduction

Key components of heavy-duty gas turbines are used in high temperature and pressure environment during long-term service, which require high surface integrity and serviceability. As a typical difficult-to-cut material, the superalloy used for these key components usually has good comprehensive properties, such as high strength, corrosion resistance, and fatigue resistance. In the machining of such hard-to-cut alloys, high cutting temperature, serious tool wear, poor quality, and other problems are often improved by various auxiliary machining methods, such as nitrogen [1], CO_2_ [2], and cooling machining, by reducing the cutting temperature to improve the surface quality [3], while the laser-assisted cutting method reduces cutting force but accelerates tool wear [4]. MQL lubrication improves surface quality, while reducing tool wear and cutting temperature, due to cooling and local lubrication of the cutting zone [5]. The cutting fluid during machining can improve the machined surface integrity, such as reducing the surface roughness [6], decreasing the strain hardening [7], etc., so the cutting fluid remains essential in the actual machining process.

In order to utilize the anti-rust properties of the cutting fluids, sometimes, the machined parts are not cleaned after machining in industrial production, and the cutting fluid that remains on the surface is in long-term contact with the parts. The polar molecules and surface-active components in the cutting fluid may cause certain corrosion and affect the service life [8]. In recent years, accidents caused by improper use of cutting fluids often occur in actual production. The impact of cutting fluids on surface quality cannot be ignored, and this impact includes both beneficial and detrimental aspects [9,10]. At present, the existing research on the effects of cutting fluid mainly focuses on the lubrication [11] and cooling properties [12,13], machined surface roughness [14,15], the development of new environmentally friendly cutting fluids [16], etc. The cooling performance of cutting fluids consists of two main aspects: one is the ability to dissipate heat, and the other is the reduction in friction, thereby reducing the heat caused by friction. It is believed that the friction and lubrication principle of the cutter-chip contact area during cutting is the capillary permeability principle, and the relevant permeability models mainly include the cuboid model of J.A. Williams [17] and the cylinder model of Godlevski [18]. J.A. Williams [19] studied that cutting fluids reduce the adhesion area between the tool and the newly formed chip surface, thus achieving the effect of lubrication. Tarek Braham-Bouchnak et al. [7] found that High Pressure Coolant (HPC) could significantly extend tool life and improve machining surface integrity in the cutting of titanium alloys. Priarone et al. [20] showed that, compared with dry cutting and traditional wet cutting on 45 steel truning, MQL lubrication could obtain better surface roughness, corrugability, surface roughness bearing ratio, and surface topography. Amigo et al. found that, for the processing of Inconel 718 superalloys, the use of oil emulsions had lower surface roughness than CO_2_ due to the higher lubrication capacity [21]. The reduction in the friction coefficient is directly related to lower machining force and surface roughness due to better cooling and lubrication [22,23]. However, in some cases, the use of cutting fluids could not improve the surface quality. Zeilmann et al. [24] found that, in the cutting of typical high temperature alloys, such as iron-nickel-chromium alloy, the chip tumor formed by dry cutting replaced the cutting edge cutting, resulting in a better surface quality than wet cutting, due to the higher temperature in the cutting area. Wei et al. [25] proposed a light lubrication milling method based on vibration signals and found that LQL stabilized the vibration at higher cutting speeds, which was beneficial to the tool and the entire cutting process. However, there are few studies on the corrosion of cutting fluid on machining surfaces, even though the corrosion has a great influence on the fatigue and tribological properties [26,27]. Therefore, it is necessary to comprehensively consider the improvement of cutting performance and corrosion characteristics of a machining surface with cutting fluid.

According to the preliminary research by our group, the main influence of cutting fluids on machined surfaces are corrosion or damage of microstructure, loss of effective metal elements, and decline of mechanical properties—especially fatigue resistance. The effect of cutting fluids on the machined surface integrity of high precision parts, such as gas turbine blades, and the effect on service life and service performance of these parts need to be studied.

In this study, the effect of cutting fluids on the milled surface quality of nickel-based superalloys was investigated in conjunction with the characteristics of different cutting fluids. The surface roughness, morphology, residual stress, and elemental compositions of the machined surfaces under different cutting fluids were investigated—especially the properties of cutting fluids. This research is of great significance for the control of machined precision and high integrity and serviceability of the subsurface layer, as well as for the selection and management of the cutting fluid during the machining of difficult-to-cut materials.

## 2. Materials and Methods

### 2.1. Workpiece Material and Cutting Fluids

**Figure 1:**
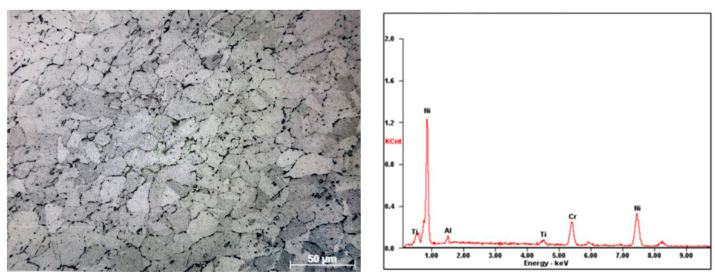
The elemental composition of NiCr20TiAl-T6.

A typical binary Ni-Cr solid solution, age-hardening, nickel-based superalloy NiCr20TiAl-T6, with a grain size of around 20 μm, was employed as the workpiece material, as shown in Figure 1, which was widely used in heavy gas turbine high-pressure rotary blades. The main composition (wt.%) of NiCr20TiAl-T6 comprised Ni ≥ 65, Cr 18.0–21.0, Ti 1.80–2.70, Al 1.00–1.80, Ti + Al ≥ 3.50, Fe ≤ 1.50, Co ≤ 1.00, and Mn ≤ 1.00, as well as trace amounts of C, Si, P, S, B, and Cu. Based on the actual machining process in industry, two universal micro-emulsified cutting fluids, denoted as E709 (Master brand, TRIM ^®^ E709 soluble oil) and Blasocut (Blaser Brand, Blasocut 4000 strong mineral oil base high performance cutting fluid), were selected and researched. In order to make sure the cooling, lubricating and corrosion properties of these two cutting fluids, the physical and chemical properties, including viscosity, friction coefficient, surface tension, cooling performance, and pH, were examined, similar to the previous research [28]. Each test was repeated three times, and then, the average was obtained.

### 2.2. Milling Experiments

Single factor milling experiments were carried out on a DMU 80 mono BLOCK (DMG, Hamburg, Germany) high-speed machine at a room temperature of about 25 °C. The diameter of the cutter head is 40 mm with four inserts. The selected insert 00005973 SECO is a PVD-coated fine grain cemented carbide with a tool nose radius of 0.8 mm. New tool noses were employed for each group of cutting tests to avoid tool wear or damage on the machining process. The cutting fluid conditions were variable, while the cutting parameters were the same as actual machining, which were fixed as follows: cutting speed *v* = 79 m/min, feed rate *f*_z_ = 50 μm/tooth, and depth of cut *a*_p_ = 0.2 mm. The cutting experiments were, respectively, carried out under the lubrication conditions with E709, Blasocut, and control test deionized water. The cutting fluids were about 5% volume fraction. The workpiece specimen’s dimension was about 30 mm × 40 mm × 50 mm, and the milled surface was 30 mm × 50 mm.

The machined surface topography and roughness were analyzed by Laser Scanning Confocal Microscope VK-X100 (KEYENCE Inc. Osaka, Japan), with the sampling area of 500 μm × 700 μm at three different positions. The three-dimensional roughness *S*a (defined as $Sa=(1/NM)\sum_{i=1}^{N}\sum_{j=1}^{M}\left|Z_{ij}\right|$) was selected to present the machined surface roughness. Surface topography was observed by field emission scanning electron microscope JSM-7001F, and 3D topography of the surface was measured by VK-X100.

The machined surface residual stress was measured by X-ray Diffractometer X-350A (HanDan stress technologies Co.Ltd, HanDan, China) with the stress-free diffraction angle of 135°. Three points were selected on each sample along the center of feed rate direction, and the stresses were measured both along the feed direction (defined as *x*-direction) and vertical to the feed direction (defined as *y*-direction). The main element contents (Al, Ni, Cr, and Ti) of the machined surface and subsurface were analyzed by JXA-8230 Electron Probe Microanalyzer (EPMA, JEOL Princeton, USA, Inc.) by line scanning on the polished cross section of the machined surface, and the scanned range was 10 μm from the surface.

### 2.3. Electrochemical Tests

Available results have indicated that the roughness, residual stress, and defects (such as micro cracks/holes) increase the corrosion of the material surfaces. In previous research, corrosion tests were taken out on the milled surfaces, while severe surface corrosion was concentrated on machined surface defects. It was difficult to define the effects of different cutting fluids on the corrosion property of the metal surfaces. Therefore, in this research, all test surfaces were polished. The dimensions of the cylindrical specimens were Φ10 × 5 mm. The main corrosion test area was a Φ10 mm circular surface, which was completely immersed in cutting fluid. The polished specimens were immersed into Blasocut and E709, with 5% volume fraction, for 0, 1, 15, 30, and 45 days. Then, the potentiodynamic polarization and electrochemical impedance spectroscopy (EIS) tests were conducted on a Model 273A electrochemical workstation (EG&G Inc., Princeton, USA). The low-frequency electrochemical signal was provided by a lock-in amplifier M5210.

The tests were processed in the NaCl solution, with a mass fraction of 3.5%, at a temperature of 25 °C. The scan rate of the electrode potential was 2 mV/s, and the scan potential range was −250~250 mV in the potentiodynamic polarization tests. The scanning frequency range was from 100 kHz to 5 MHz in the electrochemical impedance spectroscopy (EIS) tests. The surface topographies of the corrosion surface were also investigated by SEM.

## 3. Results and Discussion

### 3.1. Physical & Chemical Properties of Cutting Fluids

The basic functions of cutting fluid are cooling, lubrication, corrosion protection, and cleaning, which directly affect the machined surface quality, service performance, and service life. The main effect of cutting fluids can be tentatively evaluated based on their physical and chemical properties.

**Figure 2:**
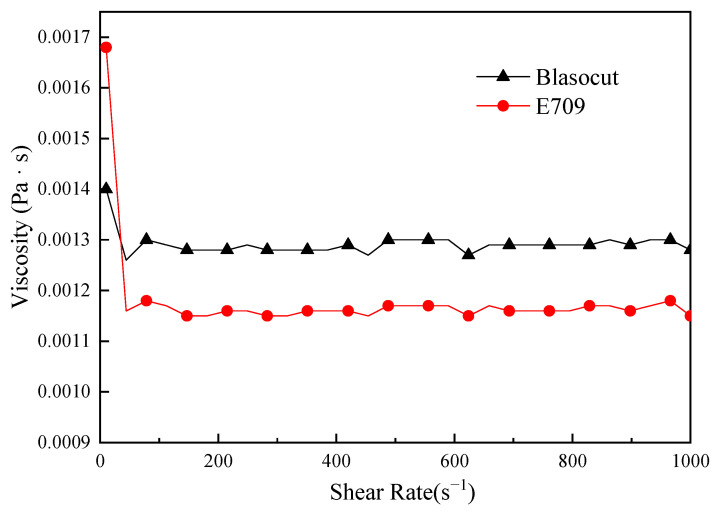
The viscosity of E709 and Blasocut.

Viscosity played an important role in the lubricating ability and permeability of the cutting fluid, which mainly affects the thickness of lubricating film. Suspensions with high viscosities are more useful when drilling metal workpieces than conventional cutting fluids [29]. The high viscosity of the cutting fluid, due to its gel strength, allows the removal of metal chips and the subsequent recovery of the slurry as a melting fluid. The viscosity was measured by the rotational rheometer (Type MCR301, Anton Paar, Antompa, Austria) at a room temperature of 25 °C, and the results were denoted in Figure 2. It shows that the viscosity of Blasocut (0.00128 Pa·s) was a little higher than that of E709 (0.00116 Pa · s). It can be speculated that Blasocut formed a stronger lubricating film than E709, and Blasocut had slightly better lubrication and wear reduction performance during the actual cutting process.

Surface tension affects the permeability of liquid, and it can be used to characterize the ability of cutting fluid to form a lubricating film. The surface tension of the cutting fluid was measured with the K-12 surface tension meter, at room temperature of 25 °C, using Wilhelmy disc method. The surface tensions of Blasocut and E709 were 34.68 mN/m and 35.13 mN/m, respectively, which indicated that the surface tensions of these two cutting fluids were with little difference, and the permeability or the speed of forming a lubricating film of them were almost the same.

**Figure 3:**
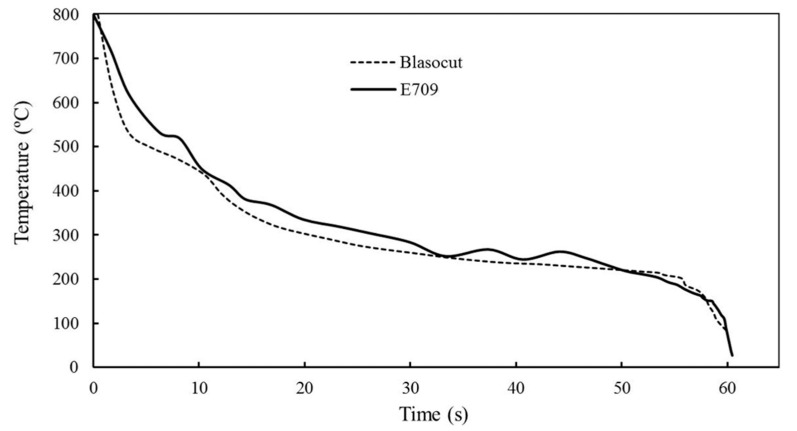
The cooling curves of E709 and Blasocut.

The cooling curves of the cutting fluids were similar to the measured results of the heat transfer coefficient of quenching oil, as shown in Figure 3. The cooling curves of these two cutting fluids were propinquity, while the cooling rate of Blasocut was larger than that of E709 in the high temperature stage (400~800 °C). As a typical difficult-to-cut material, there is an amount of heat during the machining of NiCr20TiAl-T6, and the cutting temperature is quite high in high-speed machining. The cooling function is very important for the processing quality and tool wear [30]. Due to insufficient cooling, the processing quality of MQL is inferior to that of oil emulsions [31], but the effect of oil emulsions can be approached by adding internal CO_2_ for cooling [32]. Therefore, the cutting fluid Blasocut might show a better cooling property in the machining of NiCr20TiAl-T6, resulting in better machined surface quality and longer tool life.

**Figure 4:**
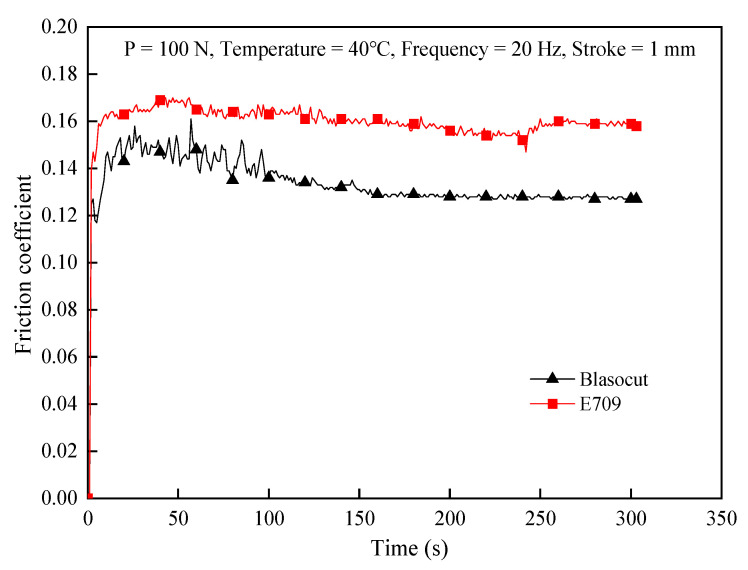
Friction coefficients of E709 and Blasocut in the friction tests.

The reduction in friction during the cutting process is one of the most important functions of cutting fluids, which can improve the machined surface quality and extend the tool life. Sliding wear tests of ball discs, under different cutting fluids conditions, were conducted on a UMT-3 environmentally controllable wear tester using a YG6X carbide ball and a 2Cr13 Martensitic heat resistant stainless-steel disk. The test temperature was 40 °C, applied load was 100 N, and the sliding frequency was 20 Hz. The friction coefficients of E709 and Blasocut in the friction tests are denoted in Figure 4. It indicates that the friction coefficients of E709 and Blasocut were 0.16 and 0.13, respectively. The lubricating property of Blasocut was better than E709. The viscosity and fluidity of cutting fluid are factors that affect the chip evacuation ability of cutting fluid, and the lubrication performance depends on the viscosity [33]. As a result, Blasocut exhibits higher viscosity and lower friction coefficient. Thus, lubrication in the cutting process is more significant.

Generally, the pH value of the cutting fluid is mainly between 8 and 10. If pH > 10, it may cause the corrosion of the operator’s skin or even the machined surface, while if pH < 8, the bacterium tend to breed and cause the deterioration of cutting fluids, which, in turn, affects the machined surface integrity. The pH value of the cutting fluids was measured with the water quality meter HANNA H9828. The pH values of E709 and Blasocut were 9.2 and 9.4, respectively, both within the limits prescribed by national standards with little differences.

According to the physical and chemical property results, the main difference between these two cutting fluids was lubrication properties, such as the reduction in friction and the ability to form a lubricating film.

### 3.2. Surface Topography and Roughness

**Figure 5:**
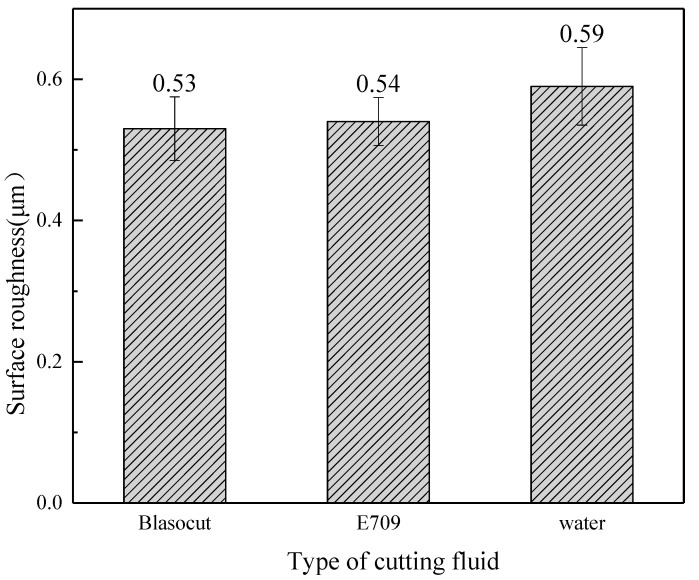
Machined surface roughness *S*a under different cutting fluids.

The three-dimensional roughness, *S*a, of the machined surfaces is illustrated in Figure 5. Cutting fluid reduces cutting forces through lubrication and cooling functions, thus reducing surface roughness [3,34,35]. It indicates that the machined surface roughness under Blasocut and E709 were almost the same, while that of water was larger (about 10%).

**Figure 6:**
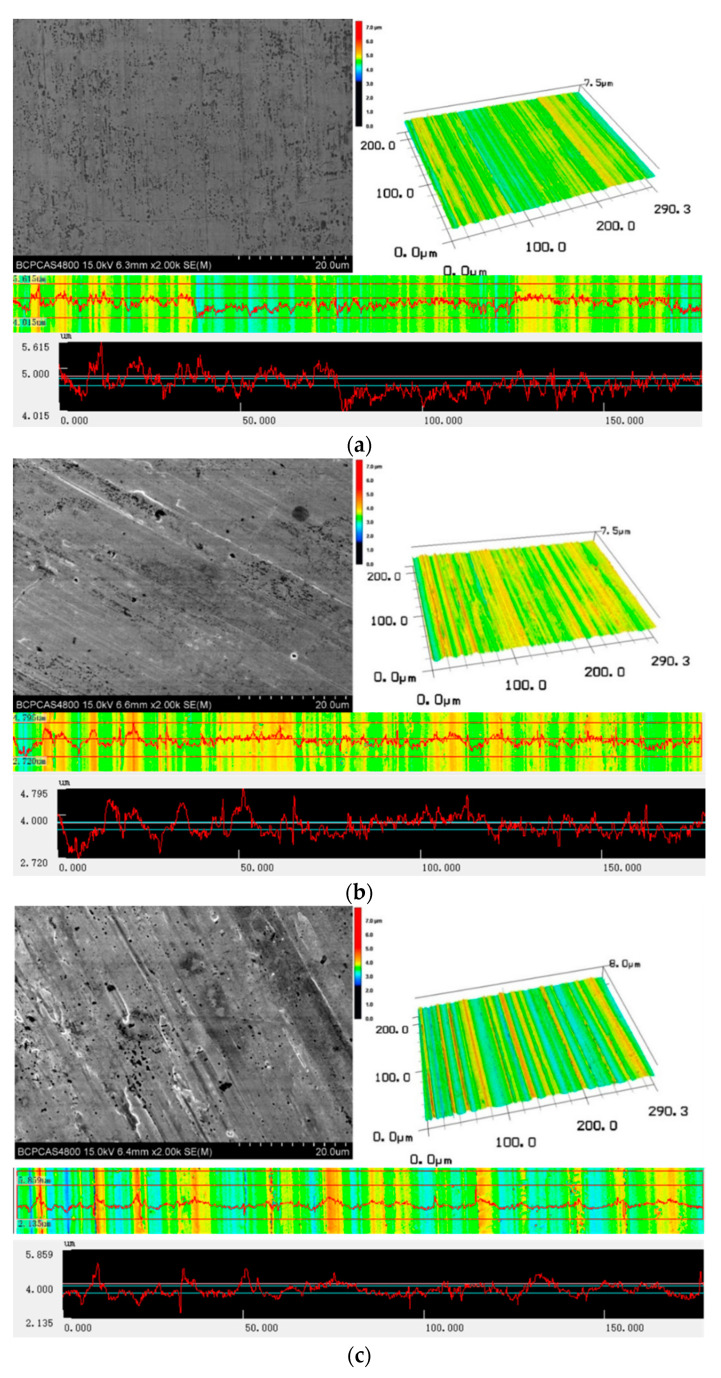
The 2D and 3D morphologies of the machined surface under different cutting fluid conditions.(**a**) Blasocut, (**b**) E709, (**c**) Water.

The SEM topographies and 3D morphologies of the machined surface are denoted in Figure 6. It was obvious that the machined surface of Blasocut was the smoothest, with almost no surface defects. The machined surface under water lubrication was the worst, with obvious gully and bulge caused by the material’s adhesive. The machined surface quality of E709 was between that of Blasocut and water. Due to the absence of oily substances in water, it was impossible to form the effective lubricating film between the tool and the material during the machining process, and there was intense friction between the tool and the material. As with the poor machinability of the Ni-based alloy, the material was easy to adhere to the tool, resulting in the poor surface finish and materials bonding. Although the machined surface roughness of E709 and Blasocut were almost the same, there were still some different defects in SEM and 3D morphologies. Due to that, the lubrication property of E709 was relatively poor, and the strength of the lubrication film formed in the cutting process was low. The failure of lubrication film in machining was the main reason for the degradation of machined surface quality.

### 3.3. Residual Stress

**Figure 7:**
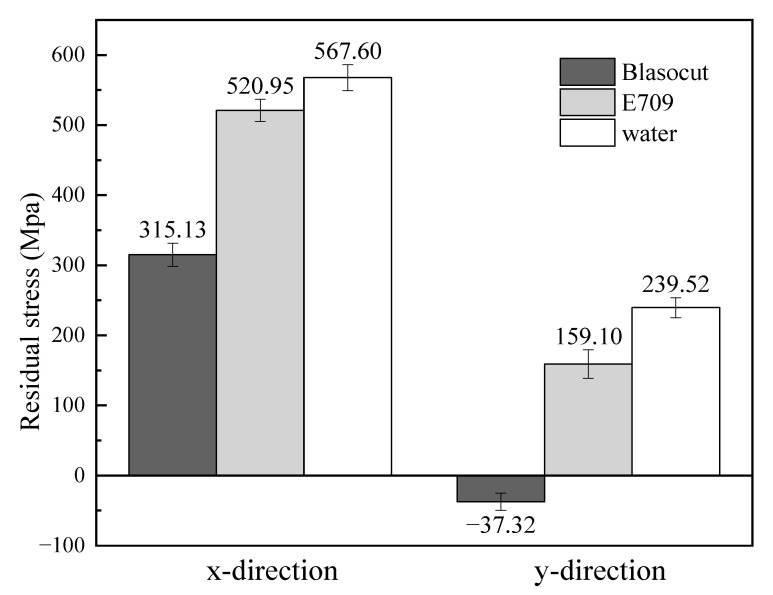
Residual stresses of the machined surface under different cutting conditions.

Residual stresses of the machined surface under different cutting fluids are indicated in Table 1 and Figure 7. It shows that the residual stresses under Blasocut, along both the x-direction and the *y*-direction, were smaller than those under E709 or water, even with residual compressive stress along the *y*-direction. The machined surface residual stresses under the action of water were larger than those of the cutting fluids.

On account of the cooling effect of the cutting fluids and the thermal stability of the materials, the machined surface residual stress of the nickel-based superalloy was mainly attributed to the mechanical and thermal effects. The material’s plastic deformation was caused by mechanical extrusion and friction between the workpiece and the tool, resulting in the surface residual compressive stress. During the cutting process, the temperature in the cutting zone raised due to a large amount of cutting heat, and the plastic deformation of the base material also occurred after the temperature decreased, resulting in the surface residual tensile stress. As mentioned above, the cooling effects of the cutting fluids were almost the same, so the difference between lubricating ability was the main reason for the disparity in machined surface residual stress under the same cutting parameters. The strength of the lubrication film of Blasocut was higher in the cutting process, as its viscosity was higher, and the friction coefficient of Blasocut was lower, so the anti-friction effect was better, which could also effectively reduce the friction heat and cutting temperature. Thus, the tensile stress caused by the thermal effect was smaller for Blasocut. The surface residual compressive stress of nickel-based alloy, after machining, is helpful to increase the fatigue life [36] For some materials, such as aluminum alloys, the initial residual stress is large and causes workpiece deformation [37,38], whereas the internal residual stress of nickel-based alloys is almost non-existent [39]. Therefore, the use of different cutting fluids directly reflects the differences in surface residual stresses and indicates that the use of Blasocut may be beneficial for fatigue life. As a new tip was employed for each test, the residual stress caused by tool geometry was close; therefore, the comprehensive surface tensile stress was small, even with compressive stress. The variation of surface roughness and residual stress, after machining with different cutting fluids, is the result of the single machining parameter. The exact effect of cutting fluid under different process conditions may vary, which will be determined in future comprehensive studies.

Existing research has indicated that the surface residual compressive stress is beneficial to improving the corrosion resistance, the fatigue life, and the creep life of the parts [40,41]. Considering the special service environment of the gas turbine blades, the residual tensile stress of the machined surface should be as small as possible in order to prolong the fatigue life and improve the service performance.

### 3.4. Surface Element Distribution

**Figure 8:**
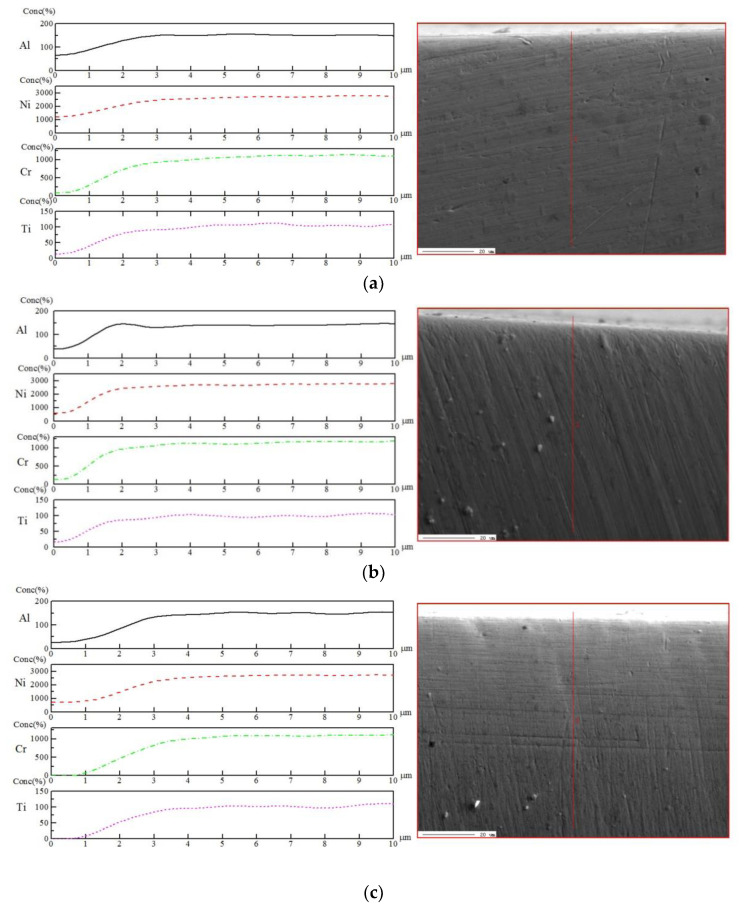
Micrographs of cross section and element contents of Al, Ni, Cr, and Ti of the machined surface under different cutting fluid conditions: (**a**) Blasocut; (**b**) E709; (**c**) Water.

The quantitative element contents of Al, Ni, Cr, and Ti varied along the cutting depth are indicated in Figure 8. It shows that the elemental contents were almost the same under different cutting fluid conditions. There was no significant change in the content of the Cr element, which was one of the most important elements affecting the micro-structure and properties of the alloy. Under the current research conditions, the machined surface element distribution was not affected by the cutting fluid conditions for the precision machining of NiCr20TiAl-T6. However, the potential effects of cutting fluids on the element loss and doping on the pre-finished surface of nickel-based superalloys were still worth noting.

### 3.5. Potentiodynamic Polarization

**Figure 9:**
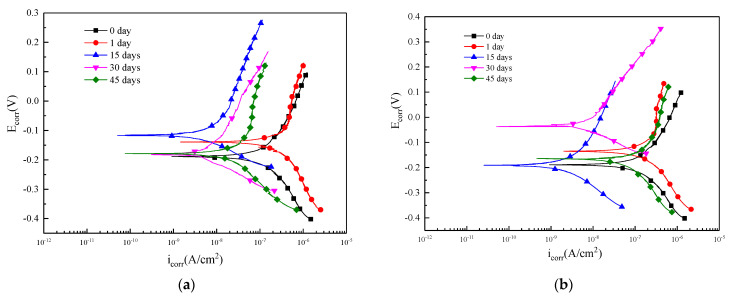
Polarization curves of specimens immersed in (**a**) Blasocut and; (**b**) E709 for different days.

Potentiodynamic polarization curve represents the relationship between electrode potential and the polarization current or polarization current density. The polarization curve was analyzed to explain the basic law of metal corrosion and reveal the mechanism of metal corrosion. The polarization curves of the samples immersed into Blasocut and E709 for 0, 1, 15, 30, and 45 days are denoted in Figure 9. It can be seen that there were obvious strongly polarized areas (Tafel areas), both in the cathodic and anode polarization curves. Additionally, the fluctuation of the polarization curves in E709 was larger than that of Blasocut, suggesting that the surface corrosion in E709 was more obvious. Then, the self-corrosion current density *i*_corr_ and self-corrosion potential *E*_corr_ of each curve were calculated according to the Tafel extrapolation method [42] shown in Table 2.

**Figure 10:**
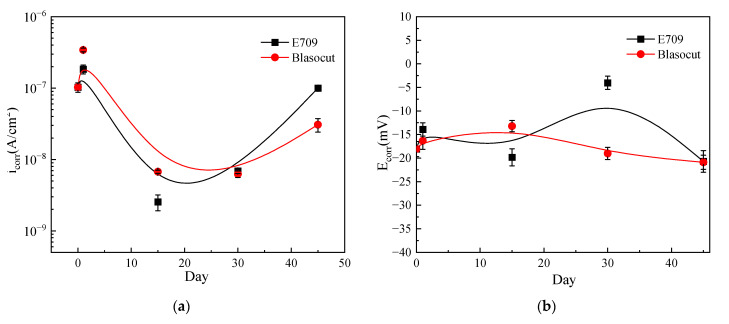
Variation of self-corrosion current density *i*_corr_ and self-corrosion potential *E*_corr_. (**a**) self-corrosion current density *i*_corr_, (**b**) self-corrosion potential *E*_corr_.

The variation of self-corrosion current density *i*_corr_ and self-corrosion potential *E*_corr_ are plotted in Figure 10. The self-corrosion potential of the specimens immersed in Blasocut changed little, only slightly increased for 15 days, and then returned to the initial level. Blasocut had a smaller effect on the material surface, and the corrosion process of the specimen was relatively gentle. For the E709 condition, the self-corrosion potential of the specimens decreased slightly for 15 days, then increased obviously for 30 days, and finally decreased for 45 days. It suggested that the corrosive properties of the specimens in E709 increased at 15 days and decreased (30 days), then increased, at 45 days. This was mainly attributed to the passivation and re-corrosion process of the metal surface.

The results of the self-corrosion current density were more obvious than the self-corrosion potential. For the specimens immersed in Blasocut, the self-corrosion current density constantly decreased with the magnitude from 10^−7^ to 10^−8^ over time, which was the corrosion rate on the surface of the sample getting smaller and smaller. At 45 days, the corrosion rate increased again, and the surface oxide film was damaged, resulting in a dispersion effect, and the surface continued to corrode. For E709, the self-corrosion current density decreased first and, then, increased. Combined with the results of the self-corrosion potential of E709, it indicated that, at the stage of decreasing the self-corrosion current density, the sample surface was passivated to form a passivation film; after the passivation film was etched, the corrosion continued and the self-corrosion current density began to increase again.

According to the self-corrosion current density icorr and self-corrosion potential, it could be found that the surface corrosion of the specimens immersed in Blasocut was very slow. The experiment results, from 0 to 45 days, represented the passivation process of the metal surface, which corresponded to the first half of the process in which the self-corrosion current density of E709 decreased. The corrosion speed of the metal surface immersed in E709 was much faster than that of Blasocut.

### 3.6. Electrochemical Impedance Spectroscopy

**Figure 11:**
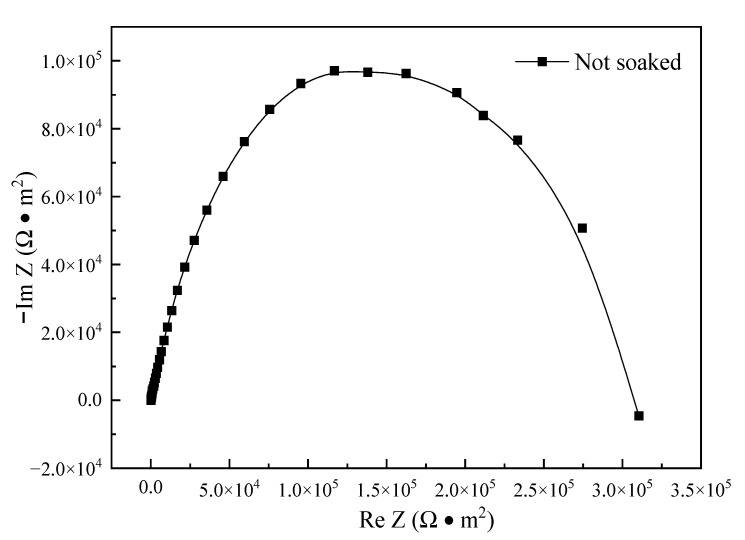
Nyquist plots of EIS for the polished NiCr20TiAl-T6 specimen.

The electrochemical impedance spectroscopy is an important tool for studying electrode process dynamics, electrode surface phenomena, and measuring the conductivity of solid electrolytes. The Nyquist plots were used to represent the impedance spectrum relation. The mechanism of the electrode process can be inferred, roughly, according to the shape of the Nyquist plots. Figure 11 shows the Nyquist plots of the electrochemical impedance spectroscopy result for the polished NiCr20TiAl-T6 specimen. This impedance spectroscopy was a single capacitive loop, which implied that there was a dense oxide film on the surface of NiCr20TiAl, and the surface was relatively stable.

**Figure 12:**
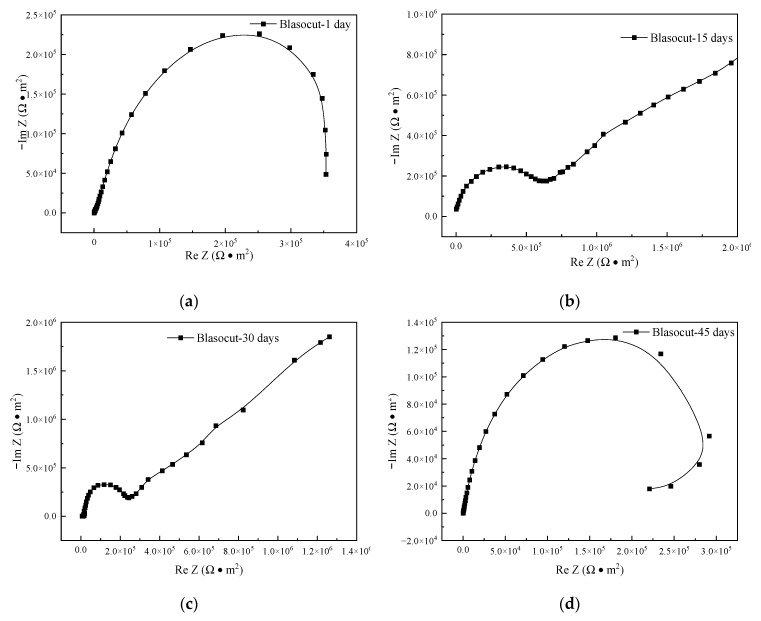
Nyquist plots of EIS for the specimens immersed in Blasocut for 1, 15, 30, and 45 days. (**a**) 1 day. (**b**) 15 days. (**c**) 30 days. (**d**) 45 days.

The Nyquist plots of the electrochemical impedance spectroscopy results of NiCr20TiAl-T6 specimens, immersed in Blasocut for 1, 15, 30, and 45 days, are indicated in Figure 12. The impedance spectroscopy of the specimen, for 1 day, was with little difference compared with the polished surface. The surface oxide film of the specimen did not change significantly, and the corrosion effect of Blasocut was not obvious in short term. The impedance spectroscopies of the specimens, for 15 days and 30 days, were approximative with the characteristic of a capacitive loop with Warburg impedance. The Warburg impedance was related to the corrosion of the oxide film on the surface of the specimens. As the immersion time was prolonged, the originally dense and stable oxide film on the surface started to be corroded, and a diffusion layer was formed.

In the impedance spectroscopies of the specimens for 45 days, there was still the capacitive reactance in the high frequency region, while the inductive reactance was in the low frequency region. The surface oxide film had been corroded, and the surface of the metal was in an unstable pitting state, also known as the pitting induction period.

**Figure 13:**
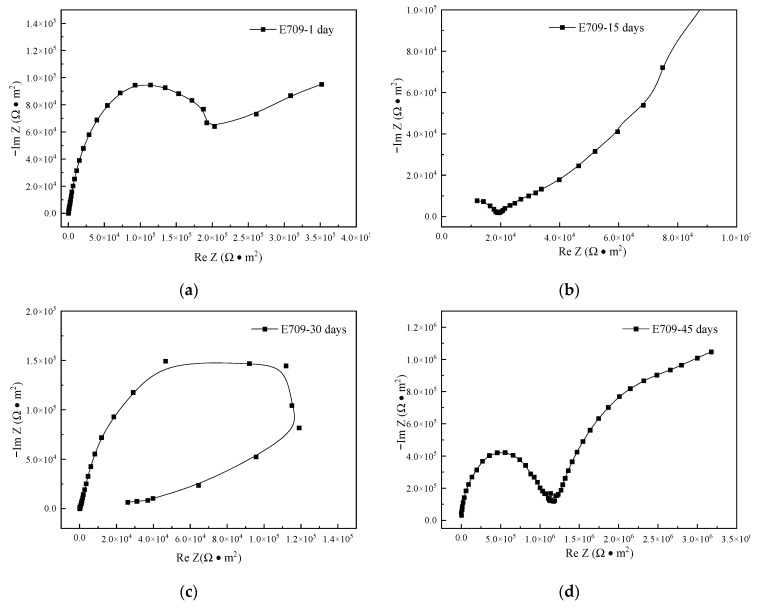
Nyquist plots of EIS, for the specimens immersed in E709, for 1, 15, 30, and 45 days. (**a**) 1 day. (**b**) 15 days. (**c**) 30 days. (**d**) 45 days.

The Nyquist plots of the electrochemical impedance spectroscopy results, for the specimens immersed in E709 for different times, are indicated in Figure 13. There was Warburg impedance in the impedance spectroscopy of the specimen for only 1 day, suggesting that the surface oxide film had started to be corroded. The Warburg impedance of the specimen for 15 days was more obvious, indicating that the surface oxide film was further corroded.

There was inductive reactance in the low frequency region for 30 days, and the metal surface was in an unstable pitting state, which was similar to that of 45 days in Blasocut. The oxide film on the sample surface had been basically corroded through, and the exposed metal matrix began to be corroded. The corrosion effect of E709 was much faster than Blasocut.

The impedance spectroscopy of the specimen in E709, for 45 days, is the key to distinguish the corrosion properties of the two cutting fluids. As can be seen in Figure 13d, there was still a capacitive loop at the high frequency region, while the capacitance characteristics were also emerged in the low frequency region. The existed research indicated that the inductive feature at the low frequency region occurred during the pitting induction period and would disappear as the corrosion increased, and the second capacitive loop occurred with the formation of real pitting holes in the corrosion surface. The EIS curve in Figure 13d illustrates that observable pitting occurred on the material surface at that time.

**Figure 14:**
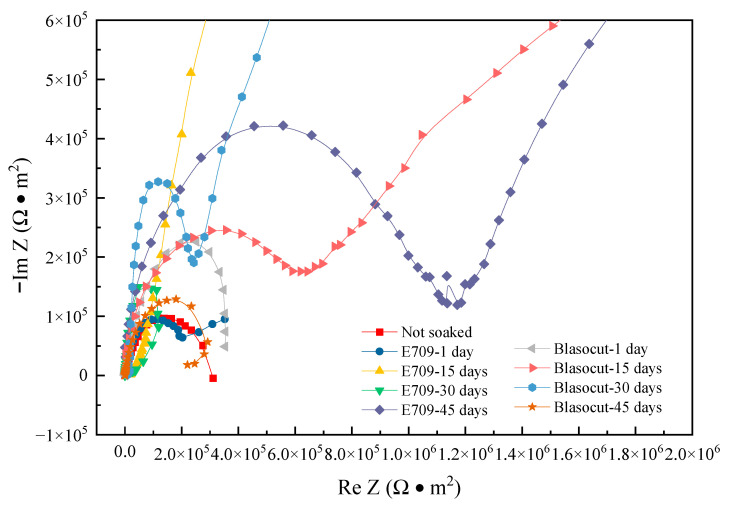
Nyquist plots of EIS for the specimens immersed in E709 and Blasocut.

All impedance spectra are represented in a diagram for analysis, as shown in Figure 14, and the fitting data of impedance spectra are shown in Table 3. In Blasocut and E709, the radius of the impedance spectrum increased first and, then, decreased as the immersion time increased, indicating that the corrosion rate decreased first and then increased, which was consistent with the variation of self-corrosion current density *i*_corr_. After 15 and 30 days of immersion in Blasocut, Warburg impedance was formed on the sample surface, indicating that, with the increase in immersion time, the originally dense and stable oxide film on the sample surface began to be corroded, forming a diffusion layer. After 45 days of immersion, the impedance spectrum radius of the specimen became smaller again, indicating that the oxide film on the metal surface was corroded, the resistance of the corrosion electrode system became larger, and the metal substrate surface was in an unstable pitting state. While the impedance spectrum of the specimen, after 1 day of immersion in E709, showed Warburg impedance, the oxide film on the surface of the material began to corrode, diffusion occurred, the radius of the resistance spectrum became slightly smaller, and the resistance of the corroded electrode system became smaller. The impedance spectrum on the 15th day of immersion exhibited a more pronounced Warburg impedance with further corrosion of the oxide film. The impedance spectrum in E709 for 30 days was similar to that in Blasocut for 45 days of immersion, the oxide film on the surface of the specimen was basically corroded completely, and it started to corrode the exposed metal substrate. At 45 days, a second capacitive arc was formed and pitting corrosion occurred on the surface. In order to prove the EIS results, micro morphologies of the corroded surfaces were analyzed by SEM.

### 3.7. Micro Morphology of Corroded Surface

**Figure 15:**
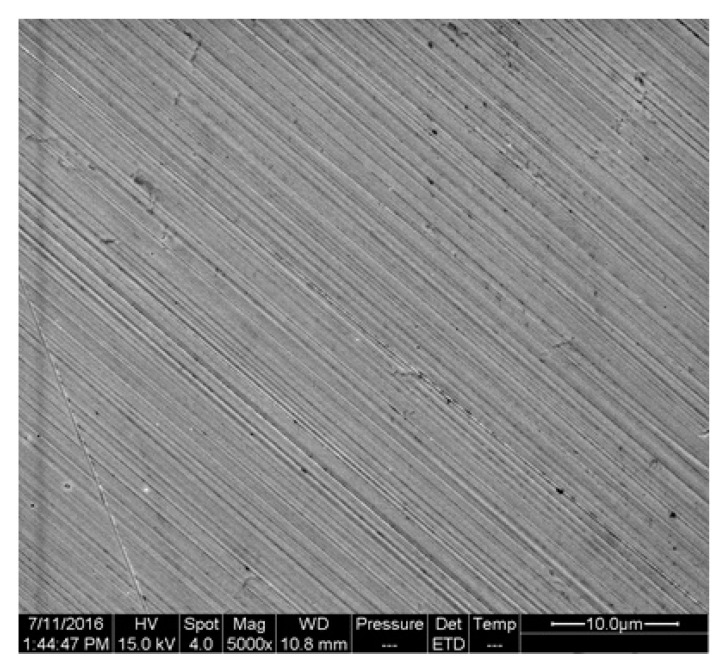
The micro morphology of polished surface.

Figure 15 indicates the micro morphology of an un-corroded polished surface. It can be seen that the surface was relatively flat, and there were no other defects except the polished texture or some hard spots.

**Figure 16:**
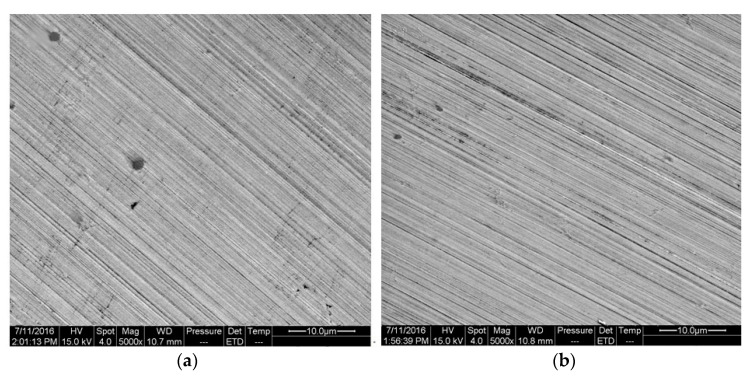
The micro morphology of specimens immersed for 15 days. (**a**) Blasocut. (**b**) E709.

Figure 16 represents the micro morphology of specimens immersed into Blasocut and E709 with 5% volume fraction for 16 days, where no obvious pits were observed. The texture of a polished surface was more obvious, which might be related to the tip discharge effect. This also supported the need to polish the sample to eliminate the interference of surface defects.

**Figure 17:**
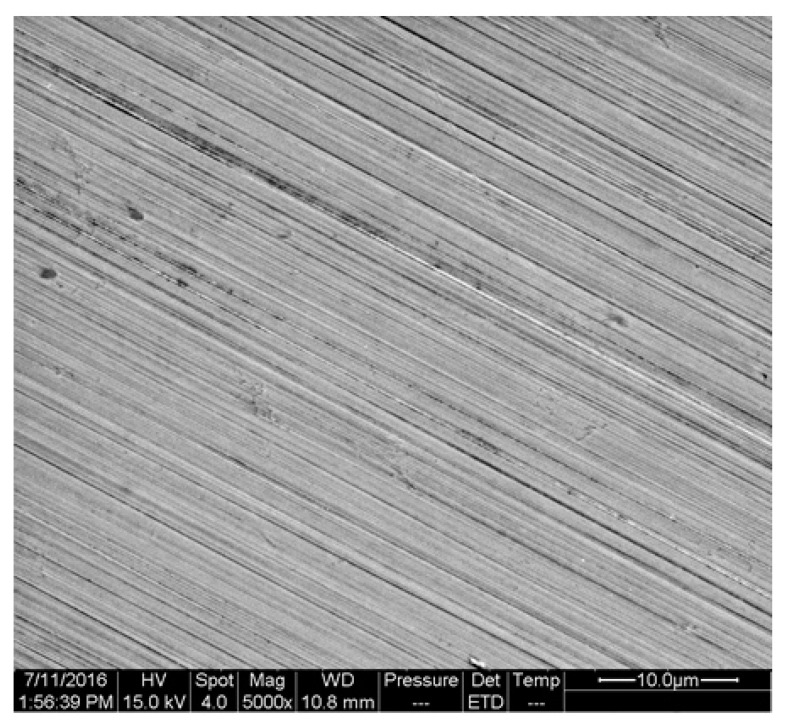
The micro morphology of specimens immersed in Blasocut for 45 days.

**Figure 18:**
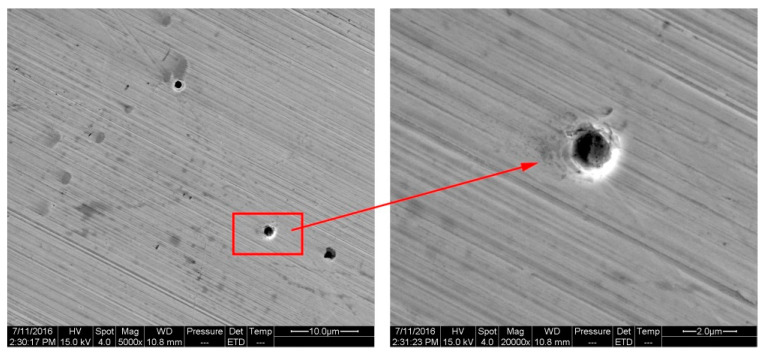
The micro morphology and the pitting corrosion hole of the surface immersed in E709 for 45 days.

The micro morphology of specimens immersed into Blasocut for 45 days are denoted in Figure 17, and it can be seen that there was no corrosive pitting, which was also consistent with the results of EIS. EIS showed that the surface immersed in Blasocut for 45 days was in unstable pitting state, and generally, the pitting corrosion could not be observed. The EIS results showed that the surface, immersed in E709 for 45 days, changed from an unstable pitting stage to a stable pitting stage, which could be observed on the surface. Some small holes were observed on the surface of the sample by SEM, as denoted in Figure 18. It can be seen that the bright circle around the small hole was caused by the layer-by-layer corrosion of metal, which accorded with the characteristics of the pitting hole. The results showed that the impedance spectroscopy could accurately predict the corrosion tendency and corrosion form of a nickel-based alloy surface, which was helpful for analyzing the matching between cutting fluid and nickel-based alloys.

## 4. Conclusions

The effects of different cutting fluids on machined surface topography, roughness, residual stress, element distribution, potentiodynamic polarization, and electrochemical impedance spectroscopy were studied for the properties of cutting fluids. The conclusions are summarized as follows:

(1) The main difference of these two cutting fluids was their lubrication property, such as the reduction in friction and the ability to form a lubricating film.
(2) The machined surface topography and roughness of E709 were between Blasocut and water. The machined surface roughness, under Blasocut and E709, were almost the same, while that of water was slightly larger.
(3) The residual stresses along both the x and y directions of Blasocut were smaller than those of E709 or water, and there were even residual compressive stresses along the y-direction.
(4) According to the self-corrosion current density and self-corrosion potential, the surface corrosion of the specimens in Blasocut was much slower than that of E709.
(5) The surface in E709, for 45 days, changed from an unstable pitting stage to a stable pitting stage with pitting corrosion.
(6) The bright circles around the small hole on the corrosion surface were caused by the layer-by-layer corrosion of the metal, which accorded with the characteristics of pitting holes.

## Figures and Tables

**Table 1:** Residual stresses of the machined surface.

Residual Stress (MPa)	Blasocut	E709	Water
*x*-direction	315.13 ± 16.5	520.95 ± 15.8	567.60 ± 18.5
*y*-direction	−37.32 ± 12.4	159.10 ± 20.3	239.52 ± 14.3

**Table 2:** Parameters of Tafel’s law calculated from polarization curves for various immersion times.

Time	Blasocut			E709		
Day	*i*_corr_ × 10^−9^ (A/cm^2^)	*E* (mV)	Corrosion Rate ×10^−4^ (mm/Year)	*i*_corr_ × 10^−9^ (A/cm^2^)	*E* (mV)	Corrosion rate × 10^−4^ (mm/Year)
0	102.8 ± 15.4	−18.07 ± 1.6	12.02 ± 1.8	102.8 ± 15.4	−18.07 ± 1.6	12.02 ± 1.75
1	342.2 ± 26.0	−16.33 ± 1.8	40.03 ± 3.0	183.9 ± 20.0	−13.91 ± 1.4	21.51 ± 2.34
15	6.730 ± 0.64	−13.22 ± 1.2	0.79 ± 0.07	2.549 ± 0.34	−19.83 ± 1.8	0.30 ± 0.04
30	6.242 ± 0.58	−19.02 ± 1.3	0.73 ± 0.07	6.917 ± 0.65	−4.020 ± 1.4	0.81 ± 0.08
45	30.75 ± 3.2	−20.89 ± 1.5	3.60 ± 0.37	99.75 ± 6.6	−20.70 ± 2.3	11.67 ± 0.77

**Table 3:** Impedance fitting results of immersion in Blasocut and E709 for different time.

Time (Day)	Blasocut				E709			
	R_s_/Ω∙cm^2^	R_1_/Ω∙cm^2^	Q_1_/μF∙cm^−2^	n_1_	R_s_/Ω∙cm^2^	R_1_/Ω∙cm^2^	Q_1_/μF∙cm^−2^	n_1_
0	12.19	2.65 × 10^4^	39.23	0.63	5.94	4.69 × 10^4^	20.98	0.63
1	13.26	4.43 × 10^7^	5.32	0.85	8.17	4.77 × 10^5^	8.28	0.73
15	5.68	3.80 × 10^5^	11.28	0.70	5.83	8.34 × 10^5^	8.22	0.76
30	3.11	3.34 × 10^7^	6.45	0.81	5.01	1.45 × 10^6^	6.28	0.77
45	12.19	2.65 × 10^4^	39.23	0.63	8.55	1.11 × 10^5^	17.5	0.67

## Data Availability

Not applicable.
